# CXCL13, CCL4, and sTNFR as circulating inflammatory cytokine markers in primary and SLE-related autoimmune hemolytic anemia

**DOI:** 10.1186/s12967-015-0474-4

**Published:** 2015-04-08

**Authors:** Boting Wu, Weiguang Wang, Yanxia Zhan, Feng Li, Shanhua Zou, Lihua Sun, Yunfeng Cheng

**Affiliations:** Department of Hematology, Zhongshan Hospital Fudan University, Shanghai, 200032 China; Department of Transfusion, Zhongshan Hospital Fudan University, Shanghai, 200032 China; Biomedical Research Center, Zhongshan Hospital Fudan Universiy, Shanghai, 200032 China; Department of Hematology, Zhongshan Hospital Qingpu Branch, Fudan Universiy, Shanghai, 201700 China

**Keywords:** Autoimmune hemolytic anemia, Systemic lupus erythematosus, CXCL13, CCL4

## Abstract

**Background:**

A considerable proportion of autoimmune hemolytic anemia (AIHA) are secondary to underlying autoimmune disorders, especially syetemic lupus erythematosus (SLE), and the clinical and laboratory index for early discrimination between primary and SLE-related AIHA has yet to be defined. In the present study, we proposed novel cytokine patterns in the pathogenesis of AIHA as well as parameters for the timely identification of SLE-related patients.

**Methods:**

AIHA patients confirmed by immunohematology techniques from September 2010 to December 2012 in our facility were consecutively included and categorized into primary (n = 19) and SLE-related (n = 18) groups. Plasma cytokine profiles were measured in a single procedure by Quantibody Human Inflammatory Array 1 (RayBiotech, Norcross, GA).

**Results:**

SLE-related AIHA patients demonstrated younger age (39 ± 20 vs.57 ± 16 years, p = 0.004), poorer reticulocyte compensation (6.8 ± 7.1 vs.12.2 ± 8.6%, p = 0.045), lower levels of lactate dehydrogenase [361 (265-498) vs. 622 (387-1154) U/L, p = 0.004], and higher occurrence of anticardiolipin antibody [9/18 (50%) vs. 2/19 (10.9%), p = 0.009]. MCP-1/CCL2, MIP-1β/CCL4, BLC/CXCL13, IL-8/CXCL8, sTNFRI, and sTNFRII were significantly up-regulated in both groups, while sTNFRII was remarkably higher in SLE-related patients. Among both groups, hemoglobin level was negatively correlated with CXCL13 (r = -0.332, p = 0.044), while reticulocyte count was positively correlated with CCL4 (r = 0.456, p = 0.005).

**Conclusion:**

CXCL13 and CCL4 could act as circulating biomarkers in AIHA, and indicated disease severity and erythroid compensation, respectively. Higher plasma sTNFRII might favor the diagnosis of SLE-related instead of primary AIHA.

## Background

Autoimmune hemolytic anemia (AIHA), classified into warm AIHA and cold agglutinin syndrome, are characterized by robust erythrocyte autoantibody production to various extents. As high as 50% of warm AIHA and 90% of cold agglutinin syndrome are considered to be secondary to infections, malignancies, and systemic autoimmune disorders including systemic lupus erythematosus (SLE), thus hindering the understanding of key immune aberrations of AIHA [1-3]. The autoantibody-centered concept for the pathogenesis of AIHA has been complemented, if not entirely altered by accumulating understandings of T helper cell and its functional subgroups. Previous studies revealed elevation of IL-10 production in both peripheral blood and spleen Th population, and suggested potential role of Treg and Th1 cells in AIHA [4-6]. However, the exact dominating Th functional population is yet to be identified in AIHA, which allows us to look for additional circulating cytokine markers that might have played potential roles in the pathogenesis of AIHA. The present study utilized a multiplex detection system to illustrate plasma cytokine portraits in primary and SLE-related AIHA patients, thus intending to identify novel inflammatory cytokine markers in this highly heterogeneous disease group.

## Materials and methods

### Study population

AIHA patients confirmed by immunohematology techniques including direct antiglobulin test and gel microcolumn antiglobulin assay from September 2010 to December 2012 in our facility were consecutively included. Patient history and baseline laboratory parameters were collected by a hematologist at the time of diagnosis. Additional panel of autoantibodies including anti-nuclear antibody, anti-cardiolipin antibody, and extractable nuclear antibodies were performed in all patients, and the diagnosis of SLE was made according to the American College of Rheumatology criteria [7]. When overt lymphadenopathy was present and lymphoid malignancies suspected, lymphadenectomy biopsy was performed to confirm or exclude the diagnosis. The health control group comprised of 10 healthy adults (6 males and 4 females) at median age of 34 (24-46) years without known history of malignancies, autoimmune diseases, or recent infections. The study was approved by Medical Ethics Committee of Zhongshan Hospital of Fudan University. Written informed consent was obtained from each patient before being included in the study.

### Plasma inflammation cytokine array

Cytokine profiles were measured by Quantibody Human Inflammatory Array 1 (RayBiotech, Norcross, GA) which permitted detection of 40 inflammation-associated cytokines including IFN-γ, I-309/CCL1, MCP-1/CCL2, MIP-1α/CCL3, MIP-1β/CCL4, MIP-1δ/CCL15, RANTES/CCL5, Eotaxin/CCL11, Eotaxin-2/CCL24, MIG/CXCL9, BLC/CXCL13, G-CSF, M-CSF, GM-CSF, ICAM-1, IL-1α, IL-1β, IL-1ra, IL-2, IL-4, IL-5, IL-6, IL-6sR, IL-7, IL-8/CXCL8, IL-10, IL-11, IL-12p40, IL-12p70, IL-13, IL-15, IL-16, IL-17, TIMP-1, TIMP-2, PDGF-BB, TNFα, TNFβ, sTNFRI and sTNFRII in a single procedure using plasma samples cryopreserved at -80°C. The protein array slides spotted by specific capture antibodies were incubated with thawed plasma samples, washed, and incubated with a cocktail of biotinylated antibodies using protocol provided by the manufacturer. The slides with bound biotin were then incubated with streptavidin-conjugated Hylite Plus 555 fluor. Relative fluorescent strength was detected by LuxScan 10 K-A Microarray Scanner (CapitalBio Corporation, Beijing, China). The actual protein concentration was obtained on the standard curve plotted via standard controls incorporated into the array.

### Statistical analyses

Analysis was performed with the SPSS 11.5 software (SPSS, Chicago, IL, USA). Data were reported as mean ± standard deviation or medians (inter-quartile ranges) for continuous variables and as frequencies (percentages) for categorical variables. Continuous variables between groups were assessed by the one-way ANOVA or Mann-Whitney U-test as appropriate. Differences in percentages were evaluated using the χ2 tests or Fisher’s exact tests. The correlation between circulating cytokine levels and clinical parameters was calculated by Pearson’s correlation coefficient or Spearman rank correlation coefficient as appropriate. Statistical significance was defined as two-sided P < 0.05. The level of significance for pairwise comparisons was adjusted when multiple comparisons were performed (p = 0.05 / 3 = 0.017).

## Results and discussion

### Clinical characteristics of primary and SLE-related AIHA

From September 2010 to December 2012, 46 patients were serologically confirmed as warm antibody AIHA in our facility, among which 18 patients were later categorized as SLE-related AIHA, 7 lymphoma-related AIHA (4 angioimmunoblastic T cell lymphomas, 2 chronic lymphocytic leukemias, and 1 nodal marginal zone lymphoma), 1 drug-induced AIHA, 1 related to non-hematological malignancy, and the rest 19 patients were categorized as primary AIHA.

SLE-related AIHA patients were significantly younger than primary cases at the time of diagnosis (39 ± 20 vs.57 ± 16 years, p = 0.004), meanwhile the serological parameters for hemolysis including lactate dehydrogenase [361 (265-498) vs. 622 (387-1154) U/L, p = 0.004], total bilirubin (1.4 ± 1.4 vs.3.1 ± 1.7 mg/dl, p = 0.002), and conjugated bilirubin (0.7 ± 0.7 vs.1.9 ± 1.3 mg/dl, p = 0.002) were less prominently elevated. Gender distribution, severity of anemia, and platelet count were similar between SLE-related and primary AIHA patients, although reticulocyte count seemed to be lower in SLE-related cases (6.8 ± 7.1 vs.12.2 ± 8.6%, p = 0.045). Except for anticardiolipin antibody [9/18 (50%) vs. 2/19 (10.9%), p = 0.009], major immunological parameters including complement levels, anti-nuclear antibody, direct antiglobulin test, indirect antiglobulin test, and peripheral lymphocyte phenotype were all comparable between SLE-related and primary AIHA patients (Table 1).

**Table 1:** **Baseline characteristics of primary and SLE-related AIHA patients**

	**Primary (n = 19)**	**SLE-related (n = 18)**	**p value**
Age, years	57 ± 16	39 ± 20	0.004^※^
Female gender	9 (47.4)	13 (72.2)	0.124
Hemoglobin, g/dl	6.9 ± 1.9	7.9 ± 2.6	0.199
Reticulocyte count,%	12.2 ± 8.6	6.8 ± 7.1	0.045^§^
Platelet count, ×10^9^/L	166 ± 104	171 ± 101	0.874
Total bilirubin, mg/dl	3.1 ± 1.7	1.4 ± 1.4	0.002^※^
Unconjugated bilirubin, mg/dl	1.9 ± 1.3	0.7 ± 0.7	0.002^※^
Lactate dehydrogenase, U/L	622 (387-1154)	361 (265-498)	0.004^※^
Complements			
C3, mg/dl	73.6 ± 31.8	56.5 ± 36.7	0.296
C4, mg/dl	9.4 ± 5.5	7.2 ± 6.7	0.451
Direct antiglobulin test			
Anti-IgG (+)	19 (100)	18 (100)	1.000
Anti-C3d (+)	15 (78.9)	17 (94.4)	0.370
Autoantibodies			
Antinuclear antibody (+)	13 (68.4)	17 (94.4)	0.110
Anticardiolipin antibody (+)	2 (10.5)	9 (50.0)	0.009^※^
Indirect antiglobulin test, titer	8 (1-32)	2 (1-4)	0.169
Peripheral lymphocyte phenotype			
CD3^+^CD4^+^,%	36 ± 20	34 ± 9	0.804
CD3^+^CD8^+^,%	33 ± 18	40 ± 11	0.294
CD16^+^CD56^+^,%	7 ± 5	7 ± 4	0.984
CD19^+^,%	21 ± 12	17 ± 7	0.365

Data are presented as mean ± SD, as number (percentage), or as median (interquartile range).

^§^p <0.05, ^※^p <0.01.

### Plasma cytokine portraits of primary and SLE-related AIHA

**Figure 1:**
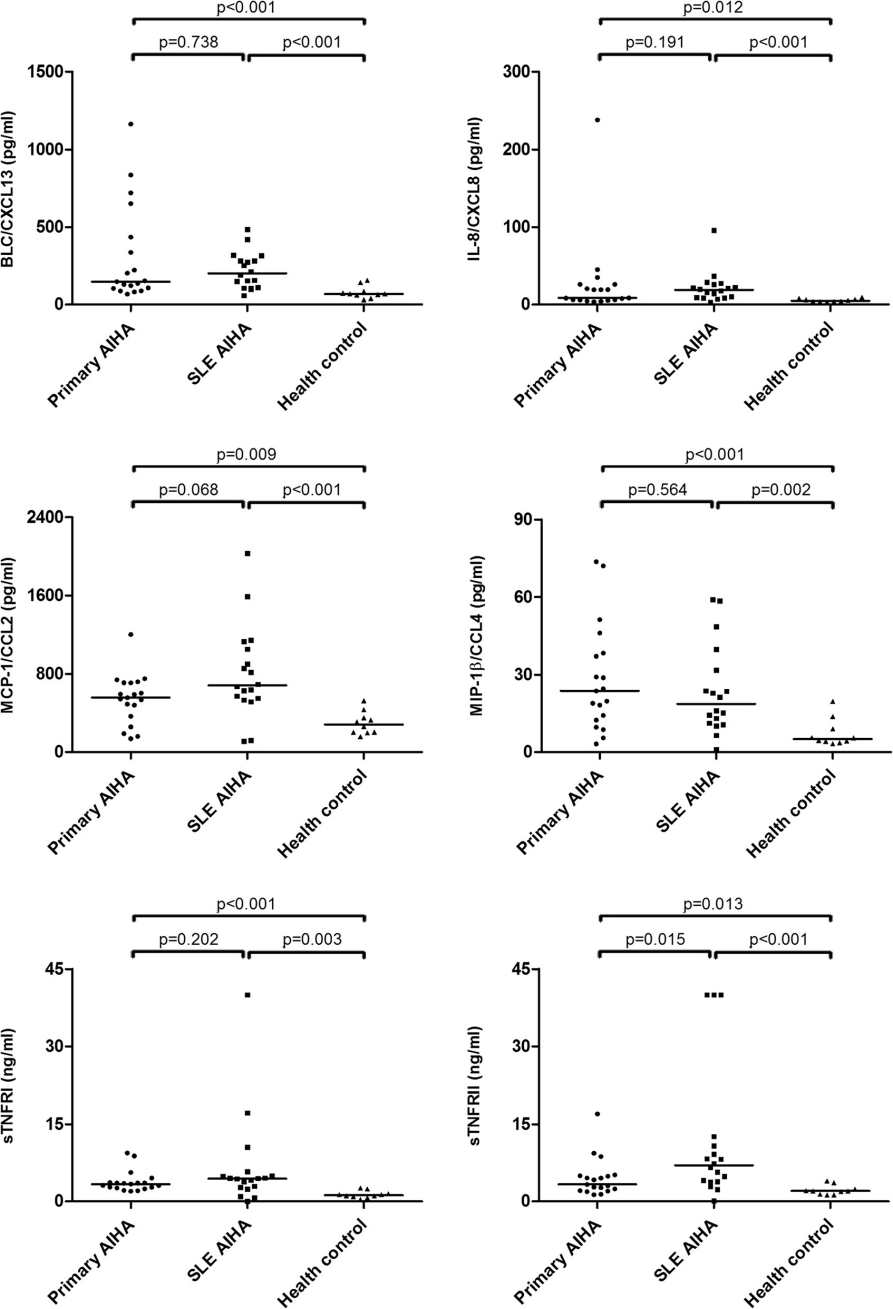
**Plasma level of CXCL13, CXCL8, CCL2, CCL4, sTNFRI, and sTNFRII in primary AIHA, SLE-related AIHA, and health control groups.** The level of significance for pairwise comparisons was adjusted when multiple comparisons were performed (p = 0.05 / 3 = 0.017).

Within the detection panel of inflammation-associated cytokines measured, MCP-1/CCL2, MIP-1β/CCL4, IL-8/CXCL8, BLC/CXCL13, sTNFRI and sTNFRII were found to be significantly up-regulated in both SLE-related and primary AIHA patients, while sTNFRII was remarkably higher in SLE-related cases. On the other hand, established cytokine markers for renowned Th functional subgroups such as Th1 (IFN-γ), Th2 (IL-4), and Th17 (IL-17) were stable, or even markedly down-regulated, in both AIHA patient groups. (Table 2, Figure 1)

**Table 2:** **Plasma cytokine portraits of primary and SLE-related AIHA patients (pg/ml)**

	**Primary (n = 19)**	**SLE-related (n = 18)**	**Health control (n = 10)**
IFN-γ	30.93 (19.53-45.66)	36.83 (26.72-69.23)	48.78 (38.10-61.94)
I-309/CCL1	65.26 (38.62-108.86)^※^	123.78 (81.56-192.06)	179.66 (96.27-194.22)
MCP-1/CCL2	557.52 (366.62-709.14)^※^	683.57 (546.29-1070.0)^※^	284.96 (200.73-372.66)
MIP-1α/CCL3	77.23 (64.74-119.83)	114.56 (78.54-162.82)	115.37 (93.05-133.28)
MIP-1β/CCL4	23.73 (12.38-38.30)^※^	18.75 (11.11-33.73)^※^	5.05 (4.02-10.22)
MIP-1δ/CCL15 (×10^4^)	0.88 (0.72-1.16)	0.55 (0.29-1.05)	1.37 (0.56-1.85)
RANTES/CCL5 (×10^3^)	4.12 (2.40-7.38)	5.89 (1.52-16.33)	5.03 (1.94-7.24)
Eotaxin/CCL11	95.41 (64.34-138.11)	118.44 (91.65-166.06)	120.81 (92.81-133.95)
Eotaxin-2/CCL24	623.34 (329.62-810.59)^※^	412.51 (282.29-720.39)	289.56 (206.08-370.20)
MIG/CXCL9 (×10^3^)	2.89 (1.95-5.15)	3.11 (2.02-10.26)	2.36 (1.82-4.12)
BLC/CXCL13	147.90 (104.11-436.02)^※^	203.45 (110.74-290.42)^※^	69.14 (56.24-100.39)
ICAM-1 (×10^5^)	1.34 (0.66-1.99)	0.60 (0.49-0.93)^※^	1.88 (0.95-2.73)
G-CSF	11.29 (4.15-24.12)	16.44 (12.83-22.74)	25.12 (14.47-28.15)
M-CSF	35.48 (19.14-66.10)^※^	82.47 (29.22-181.19) ^#^	122.47 (71.49-150.85)
GM-CSF	36.70 (23.65-48.62)	41.60 (25.59-78.01)	48.22 (39.60-72.10)
IL-1α	7.51 (3.00-26.10)^※^	27.28 (7.85-60.07)^#^	42.77 (24.27-53.30)
IL-1β	63.98 (40.83-159.84)	133.24 (89.85-223.31)^#^	203.08 (98.42-223.41)
IL-1ra	202.27 (121.20-368.22)	416.94 (192.30-563.72)	121.82 (89.31-196.76)
IL-2	9.04 (5.14-12.20)	9.81 (6.83-18.59)	11.12 (9.43-13.68)
IL-4	8.98 (6.04-16.53)	25.87 (8.61-67.04)^#^	29.64 (13.45-35.24)
IL-5	67.17 (46.65-116.94)^※^	104.56 (55.58-155.07)	150.04 (103.54-176.69)
IL-6	52.20 (40.23-76.39)	71.41 (40.80-106.07)	62.45 (52.92-72.92)
IL-6sR (×10^3^)	4.05 (3.49-4.70)	4.31 (3.45-5.17)	4.57 (3.78-4.86)
IL-7	72.16 (44.68-115.49)	85.55 (59.18-147.61)	81.55 (72.15-110.65)
IL-8/CXCL8	8.66 (5.81-26.17)^※^	19.15 (9.23-26.68)^※^	4.84 (3.95-7.21)
IL-10	26.68 (17.98-42.12)	37.50 (19.68-71.93)	15.22 (14.40-26.26)
IL-11	205.97 (61.19-342.55)^※^	324.66 (174.49-546.81)^※^	666.37 (487.65-861.28)
IL-12p40	301.15 (83.39-558.00)	570.79 (234.36-1207.2)	277.58 (151.29-403.68)
IL-12p70	4.96 (2.52-9.90)^※^	16.41 (4.91-27.76)^#^	17.04 (10.93-22.00)
IL-13	6.82 (4.40-9.99)^※^	10.18 (5.87-14.10)	10.87 (10.53-14.63)
IL-15	149.92 (61.75-478.57)	130.10 (87.61-216.20)	194.64 (169.31-233.75)
IL-16	127.04 (87.19-188.99)	187.65 (105.95-365.92)	168.00 (102.51-214.64)
IL-17	89.62 (36.65-197.82)^※^	184.42 (78.67-298.60)^※^	438.99 (279.09-667.12)
TIMP-1 (×10^4^)	1.15 (0.87-1.46)	1.63 (0.77-3.29)	2.18 (1.13-3.67)
TIMP-2 (×10^4^)	1.62 (1.37-1.78)	1.97 (1.40-2.56)	1.97 (1.66-2.26)
PDGF-BB (×10^3^)	2.45 (1.79-2.83)	2.39 (1.99-3.00)	1.56 (1.44-2.00)
TNFα	57.42 (31.45-106.25)	85.66 (36.50-121.91)	104.09 (87.59-148.24)
TNFβ	28.24 (26.75-71.80)^※^	46.24 (27.84-128.56)	92.78 (64.99-144.54)
sTNFRI (×10^3^)	3.41 (2.63-3.68)^※^	4.43 (2.67-5.18)^※^	1.21 (0.89-1.72)
sTNFRII (×10^3^)	3.30 (2.12-4.99)^※^	6.99 (3.84-11.24)^※#^	2.05 (1.37-2.70)

Data are presented as median (interquartile range). The level of significance for pairwise comparisons was adjusted when multiple comparisons were performed (p = 0.05 / 3 = 0.017).

^※^p <0.017 compared to health control; ^#^p <0.017 compared to primary AIHA.

### Correlation between plasma cytokines and clinical manifestation in AIHA

**Figure 2:**
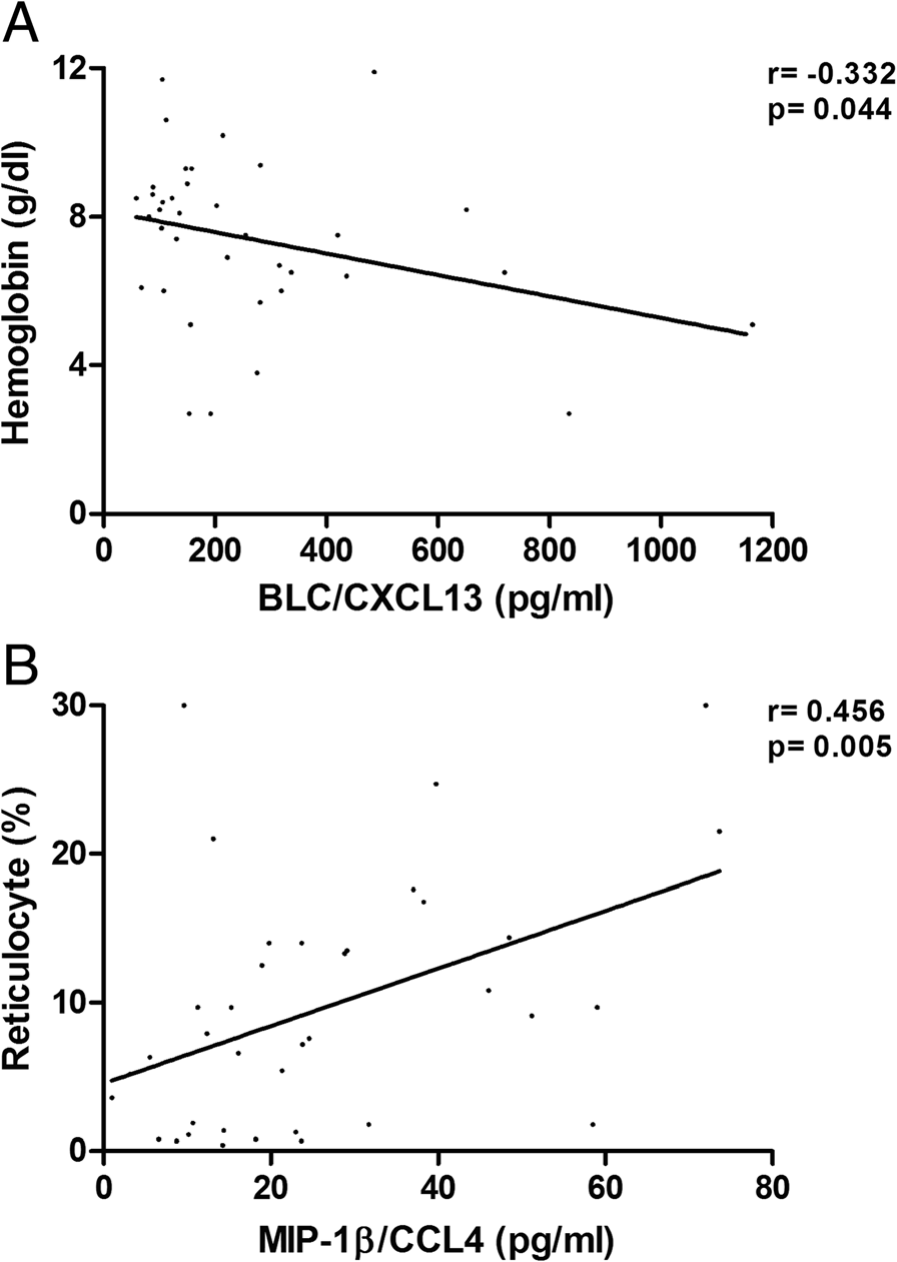
**Correlation between hemoglobin and plasma CXCL13 level (A), reticulocyte count and plasma CCL4 level (B), among both primary and SLE-related AIHA patients.**

Correlation analyses between plasma cytokine levels and clinical parameters including hemoglobin level, reticulocyte count, and lactate dehydrogenase were performed among 37 AIHA patients of both primary and SLE-related genre (Figure 2). Among the significantly up-regulated cytokines, CXCL13 was found to be negatively correlated with hemoglobin level (r = -0.332, p = 0.044), while positive correlation was found between CCL4 and reticulocyte count (r = 0.456, p = 0.005).

## Discussion

As one of the earliest acknowledged autoimmune disorders, warm antibody AIHA occurs in 1.25-2.6:100,000 persons per year [1,2]. The diagnostic panel of AIHA is mainly based upon immunohematological methods developed since mid 20^th^ century. Our understanding of the pathogenesis of AIHA is still limited except for its high prevalence of autoantibody. Fagiolo *et al.* reported enhanced IL-10 but decreased IL-12 production among peripheral blood mononuclear cells from primary AIHA patients, which indicated IL-12/IL-10 imbalance and possible Th1/Th2 dysregulation [4]. However, the repeatedly observed IL-10 upregulation was later attributed to regulatory T cells for Th1 inhibition, and the key Th functional subgroup was argued to be Th1 instead of Th2 or Th17 [5,6]. In the present study, we found 2.1- and 2.9-fold elevation of plasma CXCL13, a specific chemokine ligand for CXCR5, among primary and SLE-related AIHA patients, respectively. Meanwhile, plasma cytokines hallmarked for established Th subgroups including IFN-γ, IL-4, and IL-17 were not found elevated in either AIHA genre. Follicular helper T cell (Tfh) has recently been acknowledged as an independent Th population characterized by surface marker CXCR5. Tfh is crucial for the formation and maintenance of germinal center, and the defect of Tfh self-tolerance may be responsible for autoimmune disorders, especially those marked with high level autoantibody production [8]. We supposed that Tfh might play an important role in the process of autoantibody production among AIHA patients, and CXCL13 could emerge as a potential circulating marker for disease severity.

CCL2, CCL4, CXCL8, and sTNFRI upregulation represented common immune aberration in addition to CXCL13 in both primary and SLE-related AIHA. Interestingly, unlike CXCL13 whose plasma level reflected severe disease, CCL4 might serve as an indicator for robust bone marrow compensation among AIHA patients. Known as macrophage inflammatory protein (MIP)-1β, CCL4 closely interacted with MIP-1α/CCL3 which had been proved to inhibit the proliferation of immature erythroid progenitors [9,10]. Therefore, elevated level of circulating CCL4 instead of CCL3 among AIHA patients might indicate a boost in their erythroid proliferation and reticulocytosis. On the other hand, CCL2 and CXCL8, both capable of inducing respiratory burst as well as being chemoattractants, had been under intensive investigation among SLE patients [11-18]. Although both cytokines were argued to be elevated in the scenario of SLE, CCL2 was regarded as an important biomarker for lupus nephritis flair [13-16], and CXCL8 for lupus associated interstitial lung diseases [17,18]. In immune-mediated hemolytic anemia canine models, CCL2 was also argued to experience marked up-regulation and to demonstrate prognostic value [19].

The most intriguing finding of the present study was the subtly different patterns of soluble TNFR between primary and SLE-related AIHA patients. Human TNFRII expressed restrictively upon certain T cell populations and a few other cell types, while TNFRI was found on almost all cell types. The binding of TNF to these two types of receptors generated completely opposite cell fates, with signaling via TNFRI favoring apoptosis and TNFRII survival [20,21]. Various defects in the TNFRII pathway, including upregulated expression of TNFRII and TNFRII shedding, had been implicated in SLE patients [22,23]. Based upon the 3.4-fold increase in plasma sTNFRII level, our data supported the hypothesis of TNFRII pathway defect in SLE-related but not primary AIHA. The reason for the elevation of plasma sTNFRI in both AIHA groups was still unclear, since neither TNFR type was expressed on erythrocytes [20].

From the clinical aspect of view, differential diagnosis between primary and secondary AIHA is often challenging, especially among those with positive antinuclear antibody. Aside from younger age and slight female gender preference, SLE-related AIHA seemed to demonstrate less prominent erythrocyte destruction as indicated by serum bilirubin and LDH levels, which could be attributed to impaired scavenging function of reticulo-endothelial system previously described in SLE [24]. Instead of hypocomplementemia which was traditionally believed to be an important diagnostic clue of SLE, the presence of anticardiolipin antibody and up-regulation of plasma sTNFRII might be of greater diagnostic value for SLE-related AIHA.

The present study should be viewed in the light of its limitations. It is usually hard to give an exact significance to lab findings concerning clinical situations of high heterogeneity such as SLE. Both disease activity and precedent therapeutic methods could strongly influence lab findings. The study population was relatively small due to low incidence rate of AIHA. The descriptive nature of the present study withheld affirmative causal deduction, and further functional studies could help to clarify the detailed mechanism of specific cytokine induced signaling in AIHA.

## Conclusions

The timely differential diagnosis between primary and SLE-related AIHA often looms as practical challenge under clinical scenario, given the key underlying immune aberration of AIHA remains unclear. The present study performed circulating inflammatory cytokine profiling among primary and SLE-related AIHA patients and revealed novel cytokine patterns in the pathogenesis of AIHA as well as parameters for clinical identification of SLE-related patients. Plasma CXCL13 and CCL4 could act as circulating biomarkers in AIHA, and indicated disease severity and erythroid compensation, respectively. Detection of anticardiolipin antibody and higher levels of plasma sTNFRII might favor the diagnosis of SLE-related instead of primary AIHA.

## Abbreviations

AIHA: Autoimmune hemolytic anemia
SLE: Systemic lupus erythematosus
Tfh: Follicular helper T cell
CCL: CC chemokine ligand
CXCL: CXC chemokine ligand
IFN: Interferon
MCP: Monocyte chemotactic protein
MIP: Macrophage inflammatory protein
RANTES: Regulated on activation, Normal T cell expressed and secreted
Eotaxin: Eosinophil chemotactic protein
MIG: Monokine induced by gamma interferon
BLC: B lymphocyte chemoattractant
G-CSF: Granulocyte colony stimulating factor
M-CSF: Macrophage colony stimulating factor
GM-CSF: Granulocyte-macrophage colony stimulating factor
ICAM: Intercellular adhesion molecule
IL: Inerleukin
TIMP: Tissue inhibitor of metalloproteinases
PDGF-BB: Platelet derived growth factor subunit B
TNF: Tumor necrosis factor
sTNFR: Soluble tumor necrosis factor receptor
